# Second allogeneic transplantation using umbilical cord blood for a patient with relapsed ALK+ anaplastic large cell lymphoma after allogeneic bone marrow transplantation in the era of ALK inhibitors: A case report

**DOI:** 10.1097/MD.0000000000025576

**Published:** 2021-04-16

**Authors:** Sumiko Saito, Haruko Tashiro, Ritsu Sumiyoshi, Takuji Matsuo, Tadashi Yamamoto, Kensuke Matsumoto, Jun Ooi, Naoki Shirafuji

**Affiliations:** Department of Hematology/Oncology, Teikyo University School of Medicine, Tokyo, Japan.

**Keywords:** ALK inhibitor, case report, stem cell transplantation, T cell lymphoma, target therapy

## Abstract

**Rationale:**

Anaplastic lymphoma kinase (ALK) + anaplastic large cell lymphoma (ALCL) is considered as a good prognosis lymphoma. However, in an extremely rare subset of patients, ALK+ ALCL with leukemic presentations is known to be chemotherapy-resistant. Although several novel therapies have been tested, the standard therapy for relapsed/refractory ALK+ ALCL has not been established yet.

**Patient concerns:**

An 18-year-old female patient who had conventional chemotherapy- and Brentuximab Vedotin (BV)-resistant ALK+ ALCL with leukemic presentation. She was successfully treated with an ALK inhibitor, crizotinib. Crizotinib induced complete remission (CR) and bridged to allogeneic bone marrow transplantation (BMT).

**Diagnosis:**

However, her ALCL relapsed on day 60 after BMT and she developed high grade fever and lymphadenopathy.

**Intervention:**

Although crizotinib was given to the patient immediately after relapse, she developed grade 3 nausea and could not continue to take it. Then, we gave alectinib to the patient, which promptly induced sustained CR without any further chemotherapy. The patient received second stem cell transplantation using umbilical cord blood with myeloablative regimen in 2^nd^ CR.

**Outcomes:**

The patient has been in CR under maintenance therapy of alectinib for more than 16 months.

**Lessons:**

Both ALK inhibitors demonstrated drastic efficacy for our patient who had chemotherapy- and BV-resistant ALK+ ALCL with leukemic presentation. Alectinib showed less gastro-intestinal toxicity than crizotinib and the patient was able to take it even at the relatively early phase of stem cell transplantation.

## 1 Introduction

Anaplastic lymphoma kinase (ALK)-positive anaplastic large cell lymphoma (ALCL) develops to younger patients and is considered as a disease with better prognosis than other peripheral T cell lymphomas.^[1]^ However, in an extremely rare subset of patients (3–4%), ALK+ ALCL with leukemic presentations is known to be chemotherapy-resistant.^[1–3]^ Novel therapies, such as Brentuximab Vedotin (BV),^[4,5]^ which is an anti-CD30 monoclonal antibody conjugated with monomethyl auristatin E, and ALK inhibitors,^[6–11]^ have been tested for relapsed/refractory ALK+ ALCL. Also, high dose chemotherapy followed by autologous stem cell transplantation or allogeneic hematopoietic stem cell transplantation (HSCT) have shown long-term benefits for these patients.

One ALK inhibitor, crizotinib, has shown great efficacy against relapsed/refractory ALK+ ALCL in USA and European countries.^[9,12]^ Another ALK inhibitor, alectinib, has been used for ALK+ non-small cell lung carcinoma (NSCLC)^[13]^ and was recently approved for use in ALK+ ALCL in Japan after promising phase II study.^[14]^ Here, we show a chemotherapy- and BV-resistant ALK+ ALCL patient with leukemic presentation who was treated with crizotinib and received allogeneic HSCT. However, shortly after HSCT, her ALCL relapsed and she was then administered alectinib without any further chemotherapy. Alectinib induced CR again. She received a 2^nd^ HSCT and has been in CR for more than 16 months under maintenance therapy of alectinib.

## 2 Case report

**Figure 1:**
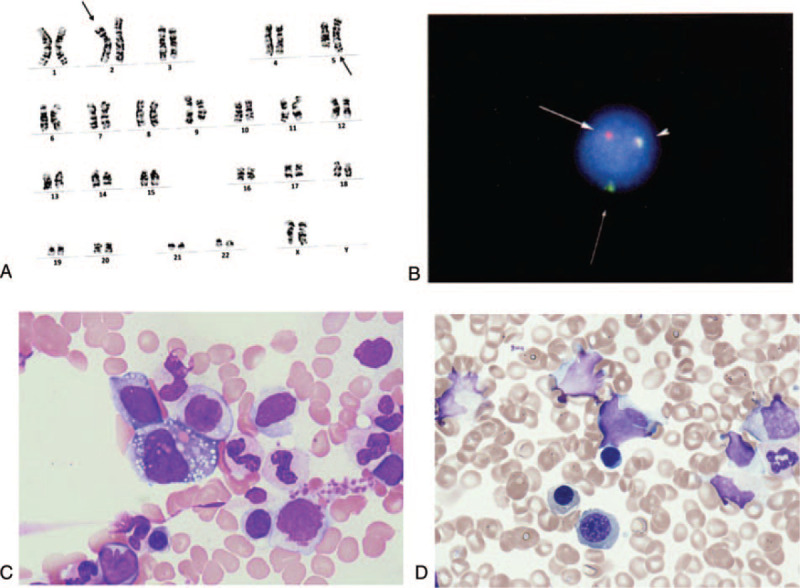
(A). Karyotype of the lymph node cells that reveals t(2;5)(p23;q35). (B). Interphase FISH analysis of the lymph node cells. ALK break-apart FISH utilizes DNA probes that hybridize to the 3’ (red signal) and 5’ (green signal) regions of the common fusion breakpoint in ALK. (C). Bone marrow smear shows infiltration of large sized atypical lymphoid cells with basophilic vacuolated cytoplasm at diagnosis (Wright–Giemsa staining x1000). (D). On day 47 of crizotinib, bone marrow smear reveals no abnormal lymphocytes infiltration (Wright-Giemsa staining x1000).

**Figure 2:**
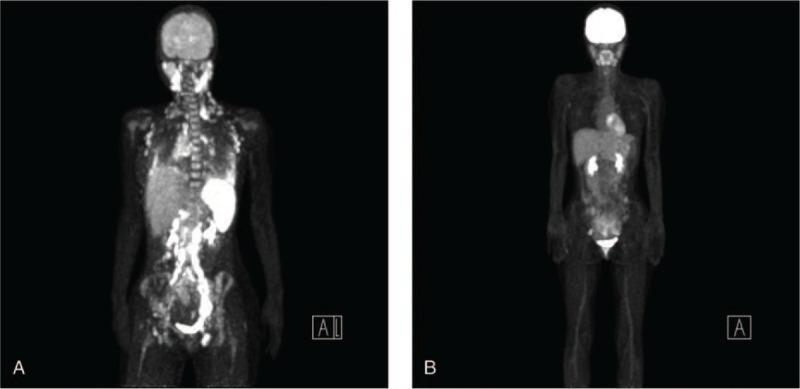
(A). PET/CT scan on day 16 of admission shows FDG uptake in systemic lymph nodes, lungs, liver, spleen, and bones. (B). PET/CT scan on day 43 of crizotinib treatment. No abnormal FDG uptake is shown.

**Figure 3:**
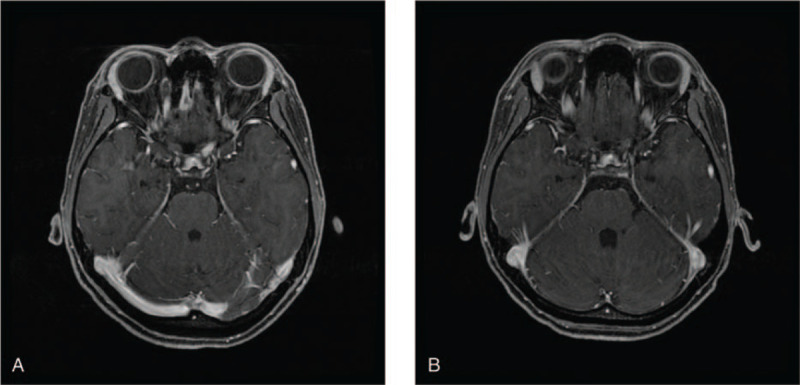
(A). On day 3 of crizotinib therapy, brain MRI shows that the enlargement of left optic nerve. (B). After 4 doses of IT and WBRT (14.4Gy), brain MRI reveals an improvement of optic nerve enlargement.

**Figure 4:**
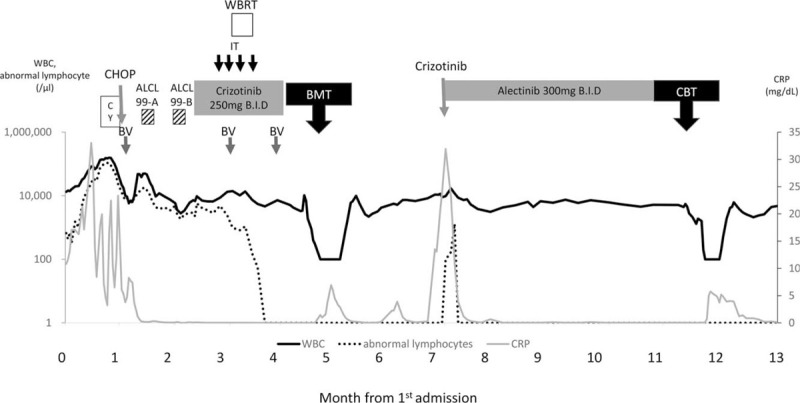
Time course of this case until 2^nd^ HSCT. The patient showed high WBC (solid line) and CRP (gray line) at admission. Although the patient received multiple chemotherapies including BV, abnormal lymphocytes (dot line) stayed in peripheral blood. Two weeks after initiating crizotinib, circulating abnormal lymphocytes started decreasing and disappeared. BV was used simultaneously twice three weeks apart. She received BMT from an HLA-matched sibling donor. In 2 months, she experienced systemic relapse that was induced to CR by alectinib. Then at 2^nd^ CR, she received CBT. ALCL99-A = dexamethasone, methotrexate, ifosfamide, cytarabine, and etoposide, ALCL99-B, dexamethasone, methotrexate, cyclophosphamide, and doxorubicin, BMT = bone marrow transplantation, BV = brentuximab vedotin, CBT = cord blood transplantation, CHOP = cyclophosphamide, doxorubicin, vincristine, and prednisolone, CRP = c-reactive protein, CY = cyclophosphamide, IT = intrathecal chemotherapy, WBC = white blood cells, WBRT = whole brain radiotherapy.

An 18-year-old female patient who did not have any prior medical history presented high grade fever, systemic lymphadenopathy, and pleural effusion. An inguinal lymph node biopsy showed an infiltration of CD30+ middle-sized lymphoid cells. The chromosomal analysis of the lymph node revealed t(2;5)(p23;q35) (Fig. 1A) that creates a fusion gene composed of nucleophosmin and ALK (NPM-ALK) and fluorescence in situ hybridization showed ALK gene translocation (Fig. 1B). Bone marrow examination was performed and showed an infiltration of middle to large sized atypical lymphoid cells with basophilic vacuolated cytoplasm (Fig. 1C). Positron emission tomography-computed tomography (PET/CT) showed fluorodeoxyglucose (FDG) uptake in systemic lymph nodes, lungs, liver, spleen, and bones (Fig. 2A). She had bilateral lung infiltration and pleural effusion that caused persistent coughing. Laboratory tests revealed a white blood cell (WBC) count of 13.3 × 10^9^/L and CRP 10.81 mg/dL at admission. On day 19 of admission, WBC count increased to 134.3 × 10^9^/L with 58% of abnormal lymphocytes and CRP 18.03 mg/dL. Given these clinical findings, we diagnosed her with stage IV ALK+ ALCL with leukemic presentation and planned to start chemotherapy with ALCL-99.^[15]^ However, after the pre-phase (dexamethasone and cyclophosphamide), she still had moderate pleural effusion. Then, we switched to CHOP (cyclophosphamide, doxorubicin, vincristine, and prednisolone) to avoid using methotrexate. CHOP therapy decreased the total number of circulating abnormal lymphocytes, shrunk the surficial lymph nodes, decreased pleural effusion, and ameliorated her fever. However, on day 8 after CHOP, her laboratory test still showed 5.9 × 10^9^/L of WBCs with 97% abnormal lymphocytes. We then gave her BV, which only decreased the ratio of abnormal lymphocytes to 20–40% in WBCs. On day 17 after CHOP, she already started developing fever and we gave her ALCL-99 course A (dexamethasone, methotrexate, ifosfamide, cytarabine, and etoposide) and course B (dexamethasone, methotrexate, cyclophosphamide, and doxorubicin) sequentially. Despite these intensive therapies, her circulating lymphoma cells did not decrease and she developed high fever. We decided to initiate ALK inhibitor therapy. At this point, neither crizotinib nor alectinib were approved for use in ALK+ ALCL in Japan. We chose to move forward with crizotinib because this drug had undergone the most thorough evaluation, with demonstrated efficacy against ALK+ ALCL in multiple studies from USA and European countries^[8–11]^ at that time. We selected crizotinib at a dose of 165 mg/m^2^ twice daily for salvage therapy as off-label use in Japan. Written informed consent from the patient and parents, and approval from the institutional committee for off-label use were obtained. Two weeks after initiating crizotinib, circulating abnormal lymphocytes started decreasing and disappeared on day 40. During crizotinib therapy, BV was simultaneously used twice three weeks apart. Although she had received 1 g/m^2^ of methotrexate as a part of ALCL regimen twice, we did not give her any intrathecal chemotherapy (IT) while detectable lymphoma cells were in circulation. Unfortunately, she developed left-sided vision disturbance right after crizotinib therapy and her brain magnetic resonance imaging (MRI) showed that the enlargement of left optic nerve (Fig. 3A). We confirmed that she had central nervous system (CNS) infiltration of lymphoma by detecting lymphoma cells in her cerebrospinal fluid (CSF). This was controlled after she received whole brain irradiation (14.4 Gray), as well as 4 doses of intra thecal (IT) chemotherapy (methotrexate, cytarabine and dexamethasone) given weekly. Since we already had the plan to perform allogeneic HSCT using 12 Gray of TBI, the dose of WBRT was reduced to 14.4 Gray. Before the 1^st^ IT, CSF cell count was 123. After second IT, it became zero. A brain MRI after 4 doses of IT and WBRT showed an improvement of optic nerve enlargement (Fig. 3B). A PET/CT scan showed no abnormal FDG uptake (Fig. 2B) and bone marrow examination did not show any lymphoma cell infiltration (Fig. 1D) on day 43 and 47 after crizotinib, respectively. She received allogeneic bone marrow transplantation (BMT) from an HLA-matched sibling donor. She was conditioned with a myeloablative regimen consisting of 12 Gray total body irradiation, 30 mg/kg etoposide, and 120 mg/kg cyclophosphamide. Crizotinib was stopped a day before the initiation of conditioning.^[10]^ As prophylaxis for acute graft-versus-host disease (GVHD), administration of 3 mg/kg of cyclosporine was started on day -1, and short-term methotrexate was given on days +1, +3, and +6. She promptly achieved engraftment with complete chimerism. Acute GVHD was not observed. Her bone marrow study did not show any lymphoma cell infiltration on day 35. She was discharged on day 37 after BMT without restarting crizotinib. However, she developed high fever with axillary lymph node swelling on day 60 and was readmitted to our hospital. A biopsy of the axillary lymph node revealed ALK+ ALCL relapse. We could not detect lymphoma cells in BM, however, the polymorphism analysis showed 7.9% of recipient pattern. Since she did not develop any neurological symptoms, we did not perform lumber puncture. Immediately after the biopsy, we started giving her crizotinib at the same dose. Within 2 days, her high fever was completely resolved. However, she developed grade 3 nausea due to crizotinib, prompting us to stop crizotinib and switch to alectinib, which is reported to have higher selectivity in ALK inhibition with decreased toxicity.^[13]^ Again, written informed consent from the patient and parents was obtained to use alectinib off-label. Alectinib was given a dose of 300 mg twice daily that did not cause any adverse events. The patient achieved CR again confirmed by CT, which was durable without any further chemotherapy for 4 months up until she received an umbilical cord transplantation. Six months after her first transplantation, she received umbilical cord blood transplantation at 2^nd^ CR using 5/8 allele-matched cord blood from a male donor. The conditioning regimen consisted of fludarabine 25 mg/m^2^ for 6 days, busulfan 3.2 mg/kg for 4 days, and melphalan 40 mg/m^2^ for 2 days. For prophylaxis of GVHD, she was given 0.03 mg/kg of tacrolimus and short-term methotrexate on days +1, +3, and +6. The number of total transplanted nucleated cells and CD34+ cells were 2.3 × 10^7^/kg and 0.8 × 10^5^/kg, respectively. She achieved engraftment promptly without developing acute GVHD. On day 40, she restarted on alectinib as maintenance therapy. She has been in CR with limited chronic GVHD for more than 16 months under alectinib treatment. She did not develop any cognitive disorder thus far. Her clinical course is summarized in Figure 4.

The patient and parents have provided informed consent for publication of this case.

## 3 Discussion

ALK inhibition demonstrated dramatic efficacy for our patient who had chemotherapy- and BV-resistant ALK+ ALCL with leukemic presentation, and successfully bridged the patient to HSCT during CR. In general, ALK+ ALCL develops to younger patients and has a good prognosis with 90% of 2-year overall survival.^[15]^ However, there are some cases that do not respond to standard therapies, especially in leukemic presentation of ALK+ ALCL which is a high mortality disease.^[1–3]^ Spiegel et al reported that only 4 out of 9 pediatric ALCL patients with leukemic presentation from a French database achieved CR by first line chemotherapy and Takahashi et al reported that only 3 out of 10 cases from the literature were alive without disease after chemotherapy, with or without HSCT.^[2,3]^ For these chemotherapy-resistant ALK+ ALCL patients, BV or ALK inhibitors have been used as a bridging therapy to HSCT.^[5,7,9,10]^

We utilized both crizotinib and alectinib during the course of this patient's treatment. Crizotinib has shown strong anti-tumor activity against relapsed/refractory ALK+ ALCL.^[10,11]^ A CR was observed in 83% of the relapsed/refractory ALK+ ALCL patients who received 165 mg/m^2^ of crizotinib and in 80% of the patients who received 280 mg/m^2^. The onset and durability of responses were not dose dependent.^[9]^ These encouraging results led us to administer crizotinib off-label to our patient and it promptly induced CR. Since the grade 3/4 neutropenia was observed in 33% and 70% of the patients who received 165 mg/m^2^ and 280 mg/m^2^ of crizotinib, respectively, we chose the lower dose for her. Alectinib is a second generation ALK inhibitor that has higher selectivity than crizotinib. In treatment-naïve ALK+ NSCLC patients, a phase III study demonstrated that the investigator-assessed progression-free survival was significantly improved with alectinib versus crizotinib.^[16]^ Unlike crizotinib, alectinib has effective CNS penetration that causes better CNS efficacy.^[17,18]^ Also, alectinib showed better tolerability than crizotinib despite the longer treatment duration in patients with Asian treatment-naïve ALK+ NSCLC patients.^[13]^ For ALK+ ALCL, there is no randomized control study to compare the efficacy and safety of alectinib and crizotinib. However, based on the studies with ALK+ NSCLC, alectinib may have therapeutic advantages for ALK+ ALCL. Indeed, our patient could not continue to take crizotinib due to severe nausea, but could take alectinib without any adverse events. She developed CNS infiltration of lymphoma while undergoing crizotinib therapy but not during alectinib therapy thus far. This may be explained by the better CNS penetration of alectinib. After a promising phase II study, Alectinib has been approved for the treatment of recurrent or refractory ALK+ ALCL in Japan.^[14]^

Tami et al showed an excellent outcome of an allogeneic HSCT in relapsed/refractory ALK+ ALCL after crizotinib with long-term remission in 5 out of 6 patients.^[6]^ The same group and others reported favorable outcomes of allogeneic HSCT in relapsed/refractory ALCL patients suggesting a graft-versus-lymphoma (GVL) effect.^[19–21]^ Although the optimal treatment for chemotherapy-resistant ALK+ ALCL patients has not been established yet, so far it seems to be a reasonable option to use novel agents to achieve remission and undergo allogeneic HSCT as soon as possible. In our case, the patient received HSCT during CR under ALK inhibitor therapy. The patient had an HLA-matched sibling donor, and we proceeded to BMT after crizotinib therapy. Shortly after BMT, her ALCL relapsed possibly indicating that there was insufficient GVL effect in this case. Since no GVHD occurred after the 1^st^ HSCT with an HLA-matched sibling donor, we did not perform the transplantation from the same donor and chose allogeneic umbilical cord blood for the 2^nd^ HSCT. The necessity of ALK inhibitor maintenance therapy after allogeneic HSCT has not been determined yet. One report in 2 ALK+ ALCL patients showed abrupt relapse after discontinuation of crizotinib.^[22]^ Although these patients were not after allogeneic HSCT, at least we should closely monitor the level of NPM-ALK after discontinuation of ALK inhibitors.

Further questions include whether allogeneic HSCT can be avoided for ALK+ ALCL in the era of ALK inhibitors. The PROFILE 1013 study demonstrated that 2-year progression free survival of 63% in relapsed/refractory ALK+ patients with crizotinib monotherapy.^[12]^ On the contrary, the long-term usage of ALK inhibitors in NSCLC patients showed resistance to the drugs.^[23,24]^ Another question is whether we should use ALK inhibitors as a first-line therapy or not. A clinical trial of combination chemotherapy with BV or crizotinib in treating patients with newly diagnosed ALCL has been conducted (NCT01979536), though the results have not yet been published.

In conclusion, both crizotinib and alectinib showed excellent efficacies against chemotherapy-resistant ALK+ ALCL with leukemic presentation. Both drugs induced CR and bridged to allogeneic HSCT. Further studies are necessary to establish the optimal strategy for relapsed/refractory ALK+ ALCL.

## Author contributions

**Conceptualization:** Haruko Tashiro, Jun Ooi.

**Data curation:** Sumiko Saito, Ritsu Sumiyoshi, Takuji Matsuo, Tadashi Yamamoto, Kensuke Matsumoto.

**Funding acquisition:** Haruko Tashiro.

**Project administration:** Haruko Tashiro.

**Supervision:** Jun Ooi, Naoki Shirafuji.

**Writing – original draft:** Sumiko Saito.

**Writing – review & editing:** Haruko Tashiro, Jun Ooi, Naoki Shirafuji.
